# Acrodermatitis dysmetabolica as a sign of methylmalonic aciduria decompensation

**DOI:** 10.1002/ccr3.1509

**Published:** 2018-04-14

**Authors:** Joana Rosa, Ana Beatriz Fraga, Rita de Carvalho, Ana Lúcia Maia, Ana Luísa Rodrigues, Maria Fernanda Gomes

**Affiliations:** ^1^ Pediatric Department Hospital of Divino Espirito Santo of Ponta Delgada, EPER São Miguel Island, Azores Portugal; ^2^ Endocrinology and Nutrition Department Hospital of Divino Espirito Santo of Ponta Delgada, EPER São Miguel Island, Azores Portugal

**Keywords:** Acrodermatitis enteropathica‐like, isoleucine, metabolic disease decompensation

## Abstract

Methylmalonic aciduria children must follow an adequate diet with low protein intake and should be regularly monitored to prevent complications. Although skin lesions like acrodermatitis enteropathica are rare in this disease, their appearance should be correlated with possible low plasma isoleucine level and it can be a sign of decompensation.

## Graphical abstract

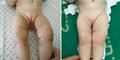

## Introduction

Acrodermatitis dysmetabolica is a designation for skin lesions resembling acrodermatitis enteropathica and observed in some metabolic disorders. We report a dermatology decompensation related to low levels of isoleucine in a methylmalonic aciduria patient. Our observation highlights the importance of maintaining an adequate supply of amino acids in protein‐restricted diets.

Methylmalonic aciduria (OMIM #251000) is an autosomal recessive disorder, caused by a defect in the conversion of methylmalonyl‐coenzyme A (CoA) to succinyl‐CoA, with consequent accumulation of metabolites of branched‐chain amino acid catabolism 1, 2. The management of these individuals consists of maintaining a protein‐restricted diet and periodic screening for possible complications 1. In recent years, skin lesions similar to acrodermatitis enteropathica (AE) were observed in organic acidurias such as methylmalonic aciduria 3, 4.

## Clinical History/Examination

**Figure 1:**
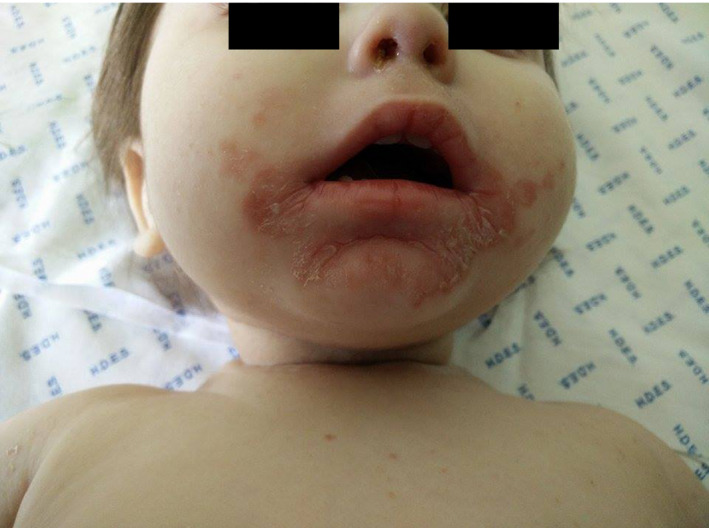
Perioral well‐defined scaly erythematous plaque.

**Figure 2:**
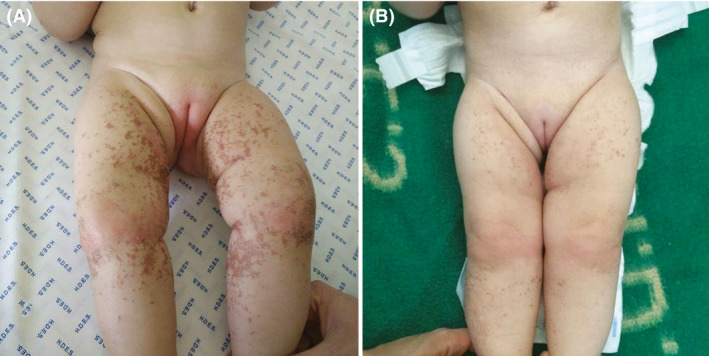
(A) Pretreatment photograph. Perineal erythema and, in the lower limbs, pinpoint to confluent erosions, associated with brownish dry crust. (B) Post‐treatment photograph showing a significant improvement with mild postinflammatory hyperpigmentation.

A 14‐month‐old Caucasian girl, with methylmalonic aciduria (MMA), mut(0) type, caused by homozygous mutation (c.682C > T), was presented to the emergency department with a maculopapular erythema in the diaper area. No fever or other symptoms, such as diarrhea or vomiting, was observed. She was treated with an ointment compound by miconazole nitrate 0.25%, zinc oxide 20%, and dexpanthenol 1.5%, admitting perianal candidiasis. Two weeks later, no improvement was observed, and the child exhibited an exacerbation of the lesions of perianal dermatitis with progression to the limbs, along with perioral scaly erythematous plaque and occipital alopecia (Figs 1 and 2A).

She had a daily diet adapted to her disease and according to the national consensus for the nutritional treatment 5: 125% energy of the recommended dietary allowance; 2 g/kg/day of total proteins; 0.8 g/kg/day of natural proteins (with essential amino acids such as isoleucine, methionine, threonine, and valine) plus a formula without branched‐chain amino acids. She had feeding difficulties with poor dietary compliance in the past weeks. She was also medicated with metronidazole 10 mg/kg/day (for 10 consecutive days each month, to reduce the intestinal synthesis of propionate, a methylmalonic acid precursor); levocarnitine 100 mg/kg/day; cyanocobalamin 1 mg/day; Vitamin D 667 IU/day; oral iron 3 mg/kg/day; probiotic and multivitamin supplement (vitamin A‐E).

She was admitted to the Pediatric Service for supervision and further investigation. Laboratory analysis revealed microcytic hypochromic anemia (hemoglobin 9.1 g/dL; mean globular volume 63.9 fL; mean corpuscular hemoglobin 19.4 pg); total proteins 5.3 g/dL; albumin 2.9 g/dL; ammonia 41 *μ*mol/L. Capillary blood gas without evidence of metabolic acidosis. The plasma level of zinc was normal 69 *μ*g/dL, and alkaline phosphatase was 162 IU/L. The results of plasma amino acids showed low levels of isoleucine: 9.1 *μ*mol/L (26.7–52.6 *μ*mol/L) and valine: 36.5 *μ*mol/L (80.2–245.7 *μ*mol/L).

Based on clinical and laboratory findings, an acrodermatitis enteropathica‐like, probably due to isoleucine deficiency, was suspected. Daily supplementation with isoleucine (40 mg/Kg/day) was started, and there was a rapid improvement of the dermatitis after four days of supplementation (Fig. 2B). During hospitalization, the protein intake was optimized (2.2 g/kg/day of total proteins and 1 g/kg/day of natural proteins). An enhancement in diet compliance was reported along with a gradual increase in isoleucine (32.9 *μ*mol/L) and valine (82.2 *μ*mol/L) values. However, after 2 years with periodic follow‐up, she still needs a diary isoleucine supplementation and one of the first signs of decompensation is perianal dermatitis.

## Discussion

The diagnosis of skin diseases can be challenging. The triad of acral dermatitis, diarrhea, and alopecia characterizes acrodermatitis enteropathica. These are associated with zinc malabsorption 3. Acrodermatitis dysmetabolica, belonging to enteropathica‐like syndrome, is a recent designation for eruptions that are similar to AE and observed in organic acidurias such as methylmalonic aciduria 3, 4. In these cases, zinc level is normal and is more likely to result from amino acid deficiency, specially isoleucine, an essential amino acid, which is responsible for keratinocytes growth 3.

Methylmalonic aciduria is a multisystem inborn error of metabolism 1. The diet of these patients consists in providing an adequate energy and avoiding endogenous protein catabolism, through low protein intake with limited doses of isoleucine, valine, threonine, and methionine 2. However, a large number of MMA children have feeding difficulties and it was reported that approximately 55% had this issue at 3 years of age 2. Therefore, lifelong follow‐up by a multidisciplinary team specialized in metabolic diseases will be required. In some cases with the persistence of a poor diet, it may be necessary to assure adequate nutrients intake through percutaneous endoscopic gastrostomy or nasogastric feeding tube 2.

In the present article, we report a child with MMA who developed acrodermatitis enteropathica‐like eruption due to inadequate isoleucine intake. Although, some cases have been reported over the years, skin manifestation in methylmalonic aciduria is not frequent 6. One of the predisposing factors was the poor dietary compliance in the weeks prior to the onset of symptoms. In the past years, in the follow‐ups with the metabolic diseases specialist, we detected that despite adequate diet according to her needs and national/international recommendations, sometimes, she has decreased isoleucine values, and one of the first manifestations is the appearance of dermatitis.

It is important to increase awareness for screening amino acid deficiency in children with cutaneous lesions receiving low protein diets, because early supplementation can reduce morbidity and the use of unnecessary therapies 7. Finally, it is also crucial to keep in mind that acrodermatitis dysmetabolica can be a sign of metabolic disease decompensation or in some cases, initial presentation 8.

## Authorship

JR: was the main author and responsible for conception, scientific research, and manuscript writing. ABF: involved in background research and article review. RC: was the nutritionist and assisted in the review and writing of the dietary component. ALM and ALR: were the doctors responsible for the follow‐up of the patient and reviewers of the article. MFG: was the responsible the Pediatric Department and reviewer of the article.

## Conflicts of Interest

The authors declare that they have no conflict of interests.

## Consent for Publication

This submission has been review and approved by all authors.
